# Hirudin inhibits ferroptosis to improve renal fibrosis by targeting the STAT3/NLRP3 signaling pathway

**DOI:** 10.1590/acb403325

**Published:** 2025-04-28

**Authors:** Fang Lan, Chunli Long, Huimin Huang, Yongxiang Xie, Wei Shi

**Affiliations:** 1 Guangxi University of Traditional Chinese Medicine – The First Affiliated Hospital – Department of Nephrology – Nanning (Guangxi) – China.; 2 Guangxi University of Traditional Chinese Medicine – School of Nursing – Nanning (Guangxi) – China.; 3 Guangxi University of Traditional Chinese Medicine – College of Graduate School – Nanning (Guangxi) – China.

**Keywords:** Hirudins, Renal Insufficiency, Chronic, Fibrosis, Ferroptosis, Signal Transduction

## Abstract

**Purpose:**

To reveal the role and underlying mechanism of hirudin in renal fibrosis.

**Methods:**

The unilateral ureteral obstruction (UUO) rat model and ferroptosis activator RSL3-induced human kidney proximal tubular epithelial cells (HK-2) were established. Hematoxylin-eosin staining, commercial kits, and immunohistochemistry were used to assess the effect of hirudin on renal function and renal fibrosis. Cell counting kit-8 assay was employed to test cell viability. Ferroptosis indicator levels were detected using commercial kits. The protein levels were examined by Western blot. The STAT3 activator colivelin was introduced to verify the role of the STAT3/NLRP3 signaling pathway in ferroptosis.

**Results:**

Hirudin alleviated renal injury and improved renal fibrosis in UUO rats. The cell viability of RSL3-treated HK-2 cells was increased after hirudin treatment. In the model group, GPX4, SLC7A11, and glutathione expression decreased, while malondialdehyde and iron content levels increased, indicating that ferroptosis was activated. Besides, p-STAT3 and NLRP3 protein levels were also upregulated. However, hirudin treatment reversed these changes. When the STAT3 activator colivelin was added, the effect of hirudin was altered.

**Conclusion:**

Hirudin improved renal fibrosis by inhibiting ferroptosis via the STAT3/NLRP3 signaling pathway.

## Introduction

Chronic kidney disease (CKD) significantly increases morbidity and mortality, and renal fibrosis is involved in its progression to renal failure^1^. When kidney injury occurs, the matrix accumulates under the action of various cells, including renal resident cells and infiltrating cells, ultimately leading to renal fibrosis^2^^,^ 3. However, there are still no targeted drugs to treat renal fibrosis.

Ferroptosis is characterized by iron accumulation and lipid peroxidation, which distinguishes it from necroptosis, apoptosis, pyroptosis, and autophagy^4^. Multiple genes regulate this cell death process, with the cystine transporter solute carrier family 7 member 11 (SLC7A11) and glutathione peroxidase 4 (GPX4) being the key negative regulatory factors^5^. GPX4 is a key antioxidant enzyme in the GPX family, which can inhibit ferroptosis by catalyzing the reduction of lipid peroxides. SLC7A11 influences the uptake of cystine to regulate GPX4^6^^,^ 7. GPX4 and SLC7A11 levels are reduced when some kidney diseases occur, suggesting that ferroptosis is activated during these diseases^8^^-^ 10. Ferroptosis potentially affects kidney disease through the induction of inflammation and exacerbation of renal fibrosis^11^^,^ 12.

Hirudin, a natural polypeptide produced by the salivary glands of blood-sucking leeches, is used diffusely in clinics due to its potent anticoagulant effect^13^^,^ 14. It has been confirmed that hirudin displays a certain role in the treatment of kidney injury^15^^,^ 16. Recent studies have also highlighted hirudin’s anti-inflammatory properties^17^^,^ 18. This effect may be associated with the suppression of NOD-like receptor thermal protein domain associated protein 3 (NLRP3) activation^19^. Chronic inflammation is a feature of renal fibrosis, damaged tubular cells, infiltrating macrophages, and myofibroblasts produce cytokines and growth factors that create an inflammatory environment when the kidneys are injured, leading to tubular atrophy and interstitial fibrosis^20^. Furthermore, the inflammatory state of proximal tubular cells significantly downregulates glutathione (GSH) metabolism genes, making the cells more susceptible to ferroptosis, and induces the accumulation of inflammatory proximal tubular cells to enhance inflammation and fibrosis^21^. In addition, NLRP3 deletion may alleviate lipopolysaccharide-induced acute kidney injury by reducing inflammation and ferroptosis^22^.

STAT3 is a multifunctional transcription factor that regulates a variety of cellular processes, including inflammation, cell proliferation, and apoptosis^23^. Studies have shown that STAT3 is activated in multiple inflammatory diseases^24^^-^ 26. The inhibition of STAT3 in diabetic nephropathy can improve renal fibrosis and inflammation^27^. Moreover, STAT3 has been proven to be a crucial regulator of NLRP3, and decreasing STAT3 expression can inhibit NLRP3 activation^28^. Luo et al. found that STAT3 promotes NLRP3 inflammasome activation by regulating NLRP3 mitochondrial translocation^29^.

Based on these findings, we hypothesized that hirudin might ameliorate renal fibrosis by alleviating ferroptosis, and that this therapeutic effect could be mediated through the STAT3/NLRP3 signaling pathway. We explored the possible mechanism of hirudin in treating renal fibrosis, providing a new therapeutic strategy for patients with renal fibrosis.

## Methods

### Animals

Thirty-five male Sprague-Dawley rats (7–8 weeks, 180–220 g) were sourced from Shulaibao Biotechnology Co., Ltd. (Wuhan, China). The rats were housed under specific pathogen-free conditions and given free access to food and water. The experimental environment was controlled with a temperature of 22 ± 2℃ and relative humidity of 45 ± 5%, as well as a 12-h light/dark cycle. After one week of acclimation for the rats, the experiments were conducted. The Ethical Committee for Animal Welfare at Yangzhou University approved these activities (Approval No: 202406023).

### Unilateral ureteral obstruction model establishment and treatment

The animals were allocated to groups randomly. Firstly, the rats were injected with 30 mg/kg pentobarbital sodium to induce anesthesia, then placed on their backs and immobilized. To establish the unilateral ureteral obstruction (UUO) model, a right abdominal incision (1.75 ± 0.25 cm) was made to open the peritoneum, exposing and separating the right ureter, which was then ligated near the renal hilum and bladder. The bowel was repositioned, and the wound was closed when there was no significant bleeding. The operation for the sham group was similar to the above procedure except that no ligation was performed. In the UUO + hirudin group, 20 IU/kg/d hirudin (MCE, HY-P2813) was injected through the tail vein^30^. In the UUO + hirudin + colivelin group, 2 mg/kg colivelin (YEASEN, 53589ES03) was administered intraperitoneally simultaneously with hirudin injection. After 14 days of treatment, the animals were euthanized with an injection of excess pentobarbital sodium intraperitoneally, and blood and kidney samples were obtained.

### Cell culture

HK-2 cells (Pricella, CL-0109) were cultured in Dulbecco’s modified eagle (DMEM) (Gibco, C11995500BT) medium, including 10% fetal bovine serum (Gibco, 10270106) and 1% penicillin–streptomycin antibiotics (Hyclone, SV30010). The cells were incubated at 37°C with 5% CO_2_. The experiment consisted of the following four groups:

- Control;
- RSL3: 1 μM RSL3 (a ferroptosis activator, MCE, HY-100218A) was used to treat the cells for 24 h^31^;
- RSL3 + hirudin: cells were incubated with 1 mg/mL hirudin for 48 h after being incubated with RSL3;
- RSL3 + hirudin + colivelin: 0.5 μM colivelin was used to treat HK-2 cells for 1 h before hirudin treatment^32^.

### Hematological analysis

Blood was collected using the retro-orbital technique and placed in 1.5-mL centrifuge tubes. The serum was isolated after resting for 1-2 h. After centrifugation, the creatinine (Crea) and blood urea nitrogen (BUN) levels were tested by an automated biochemical analyzer (HITACHI, 7020).

### Hematoxylin-eosin staining and Immunohistochemistry

The kidneys from rats were fixed and then made into sections about 4-µm thick. The kidney sections were dewaxed and hydrated, followed by staining with a hematoxylin-eosin (HE) staining kit (Beyotime, C0105S)^33^. A microscope (Olympus, BX53) was used to image these sections.

Rat kidney sections were dewaxed and hydrated for the immunohistochemistry experiment, and treated with citrate antigen retrieval solution (Beyotime, P0083) under high pressure for 1-2 min to repair antigens. The sections were soaked in a 3% H_2_O_2_ (Sinopharm, 10011208) solution for 20 min and then incubated with 50 μL of α-smooth muscle actin (α-SMA, 1:100, Affinity, AF1032) and fibronectin (1:100, Affinity, AF5335) overnight. Then, the sections were treated with 50 μL of secondary antibody working solution (1:2,000, Abcam, ab205718) for 10–15 min. Subsequently, 3,3’-diaminobenzidine (DAB, Beyotime, P0202) was added, and the sections were placed in a wet box. Finally, following staining with hematoxylin and dehydration, a light microscope was used to image the sections.

### Cell counting kit-8 assay

According to the instructions, HK-2 cells were treated with cell counting kit-8 (CCK-8) solution (Beyotime, C0037) for 2 h, and the absorbance at 450 nm was measured.

### Western blot

Protein samples were prepared from tissue homogenates, and the concentrations were measured. The proteins were transferred onto polyvinylidene fluoride membranes (Beyotime, FFP24) after electrophoresis. Subsequently, 5% skim milk (Beyotime, P0216-300 g) was used to block the proteins. Then, proteins were incubated with the primary antibodies against GPX4 (1:1,000, Affinity, DF6701), SLC7A11 (1:1,000, Affinity, DF12509), STAT3 (1:1 000, Affinity, AF6294), p-STAT3 (1:1,000, Affinity, AF3293), NLRP3 (1:1,000, Affinity, DF7438) and GAPDH (1:1,000, CST, 2118) overnight. Next, the proteins were treated with a secondary antibody (1:2,000) at 37°C under dark conditions. Enhanced chemiluminescence (Applygen, P1000) was used to visualize the protein bands.

### Antioxidant biomarker and iron content determination

Following the manufacturer’s instructions, malondialdehyde (MDA) and GSH levels were determined by MDA (Solarbio, BC0025) and GSH (Solarbio, BC1175) assay kits. Iron content in rat tissues and HK-2 cells was detected using a tissue iron assay kit (Nanjing Jiancheng, A039-2-1) and a cell total iron colorimetric test kit (Elabscience, E-BC-K880-M), respectively.

### Statistical analyses

All data are exhibited as mean value ± standard deviation. One way analysis of variance was employed to evaluate the differences. A *p* < 0.05 indicated statistical significance.

## Results

### Hirudin alleviated renal injury and improved renal fibrosis in unilateral ureteral obstruction rats

**Figure 1:**
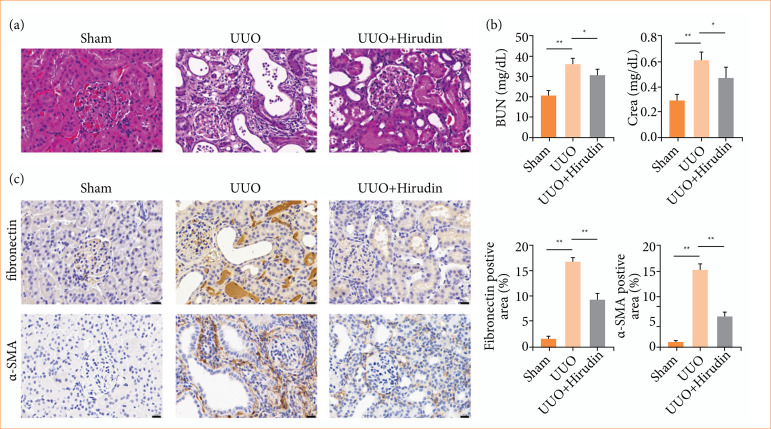
Effect of hirudin on renal injury and renal fibrosis in UUO rats. **(a)** The status of rat renal tissue was assessed using hematoxylin and eosin staining (bar = 20 μm). **(b)** BUN and Crea levels were tested by an automated biochemical analyzer (n = 5). **(c)** α-SMA and fibronectin levels were detected by immunohistochemistry (n = 3).

UUO: unilateral ureteral obstruction; BUN: blood urea nitrogen; Crea: creatinine; *p < 0.05; **p < 0.01. Source: Elaborated by the authors.

To verify the therapeutic effect of hirudin on renal fibrosis, an in-vivo UUO-induced renal fibrosis model was established. There were no abnormalities in the glomeruli, tubules, and interstitium of sham rats (Fig. 1a). However, UUO rats showed dilated renal tubules, significant glomerular hyperplasia, interstitial edema, and infiltration of inflammatory cells (Fig. 1a). Serum BUN and Crea levels were measured using commercial kits to assess renal function. The results showed that BUN and Crea levels were significantly increased in UUO rats compared to controls (Fig. 1b). Immunohistochemistry was used to test renal fibrosis marker (α-SMA and fibronectin) levels. In the model group, α-SMA and fibronectin levels were significantly higher than those in the sham group (Fig. 1c). The above findings indicated that the UUO rat model was successfully established. However, kidney injury in UUO rats was alleviated after hirudin treatment (Fig. 1a). BUN, Crea, α-SMA, and fibronectin levels were also markedly reduced (Figs. 1b and 1c), demonstrating that hirudin has a therapeutic effect on improving renal injury and renal fibrosis.

### Hirudin inhibited ferroptosis and the STAT3/NLRP3 signaling pathway in UUO rats

**Figure 2:**
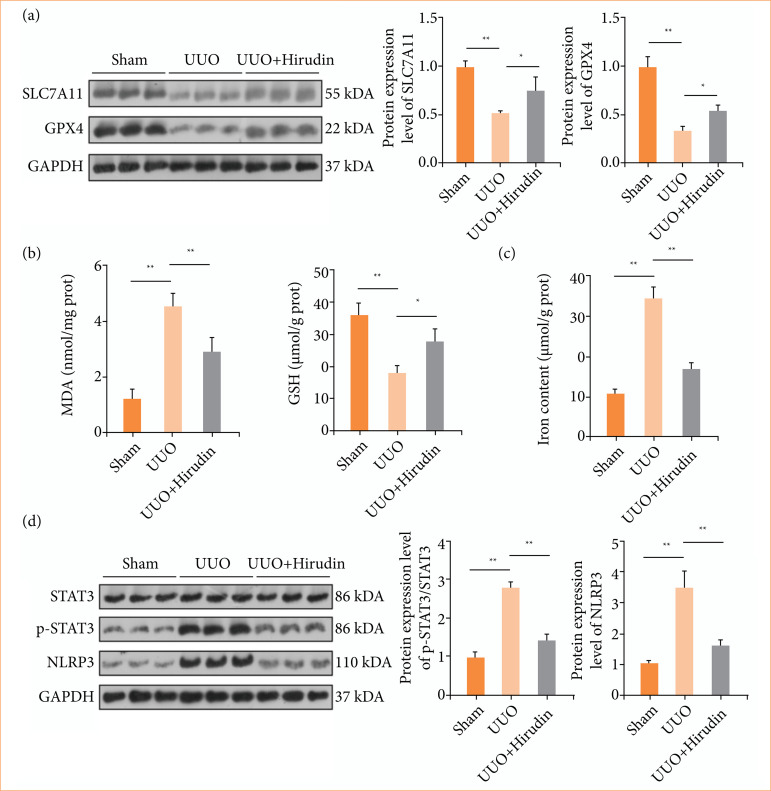
Effect of hirudin on ferroptosis and STAT3/NLRP3 pathway in UUO rats. **(a)** Protein levels of SLC7A11 and GPX4 were detected by Western blot (n = 3). **(b)** The contents of MDA and GSH were evaluated using commercial kits (n = 3). **(c)** A tissue iron assay kit was used to determine the iron content (n = 3). **(d)** Protein levels of p-STAT3 and NLRP3 were detected by Western blot (n = 3).

UUO: unilateral ureteral obstruction; MDA: malondialdehyde; GSH: glutathione; *p < 0.05; **p < 0.01. Source: Elaborated by the authors.

Ferroptosis is a significant contributor to renal fibrosis, with GPX4 and SLC7A11 serving as key markers, detected via Western blot. GPX4 and SLC7A11 levels were observably reduced in the UUO model group compared to controls, while hirudin treatment increased their levels in UUO rats (Fig. 2a). Additionally, oxidative stress marker (MDA and GSH) and iron levels were assessed by commercial kits. The results indicated that MDA and iron levels in UUO rats were markedly higher than those in sham rats, but notably reduced by hirudin treatment, while GSH showed the opposite trend (Figs. 2b and 2c). These findings indicated that hirudin can alleviate ferroptosis *in vivo*.

Moreover, the expression of NLRP3 is implicated in ferroptosis, and STAT3 acts as a crucial regulator of NLRP3^22^^,^ 28. In this study, p-STAT3 and NLRP3 levels in kidney tissues were assessed using Western blot. The results showed that p-STAT3 and NLRP3 levels were significantly elevated in UUO rats compared to controls, and hirudin treatment alleviated these changes (Fig. 2d). p-STAT3 and NLRP3 levels were associated with the activation or inhibition of ferroptosis, suggesting that the effect of hirudin on renal fibrosis by inhibiting ferroptosis may be related to the STAT3/NLRP3 signaling pathway.

### Hirudin inhibited the STAT3/NLRP3 signaling pathway to alleviate ferroptosis in RSL3-induced HK-2 cells

**Figure 3:**
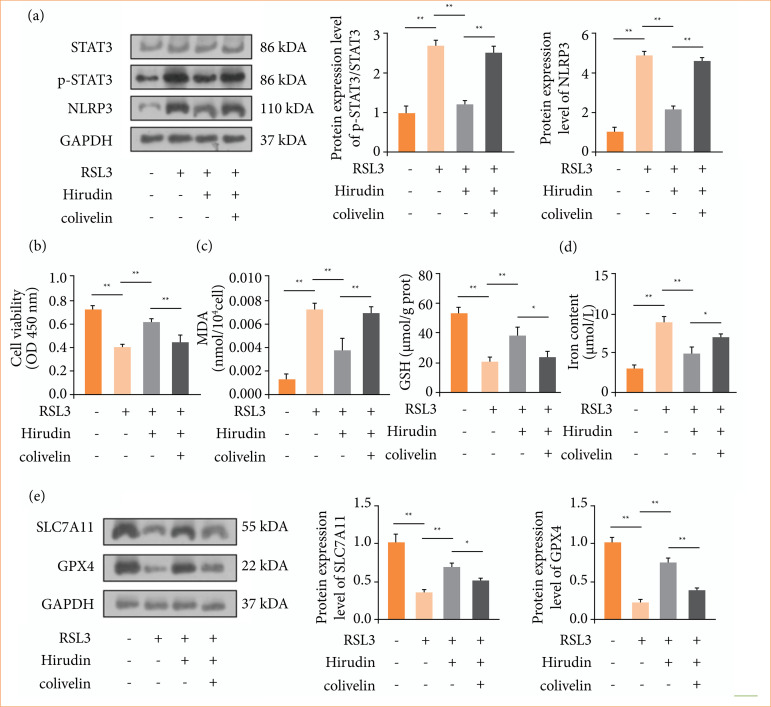
Hirudin alleviated ferroptosis by inhibiting the STAT3/NLRP3 signaling pathway in RSL3-induced HK-2 cells. **(a)** Protein levels of p-STAT3 and NLRP3 were detected by Western Blot (n = 3). **(b)** CCK-8 assay was used to assess cell viability (n = 4). **(c)** The contents of MDA and GSH were evaluated using commercial kits (n = 3). **(d)** A cell total iron colorimetric test kit was used to determine the iron content (n = 3). **(e)** Protein levels of SLC7A11 and GPX4 were detected by Western blot (n = 3).

UUO: unilateral ureteral obstruction; MDA: malondialdehyde; GSH: glutathione; CCK-8: cell counting kit-8; *p < 0.05; **p < 0.01. Source: Elaborated by the authors.

To explore the mechanism of hirudin in treating renal fibrosis, RSL3 was used to induce ferroptosis in cells, and colivelin, a STAT3 activator, was added to evaluate the role of the STAT3/NLRP3 signaling pathway in ferroptosis. Western blot results demonstrated that p-STAT3 and NLRP3 levels were significantly elevated in RSL3-induced HK-2 cells compared to controls, but they decreased after hirudin treatment. However, the effect of hirudin was reversed by colivelin (Fig. 3a), indicating that STAT3 was activated after colivelin intervention. CCK-8 assay demonstrated that RSL3 treatment markedly decreased cell viability in HK-2 cells (Fig. 3b). However, hirudin increased the viability of HK-2 cells in the RSL3 group, whereas it was reduced following colivelin treatment (Fig. 3b). Hirudin reduced cell death induced by ferroptosis *in vitro*, while activation of the STAT3/NLRP3 signaling pathway counteracted its effects.

Antioxidant biomarkers (MDA and GSH) and iron content were determined using commercial kits. As shown in Figs. 3c and 3d, MDA and iron levels were significantly elevated after RSL3 intervention, but they decreased following hirudin treatment, while GSH showed an opposite trend. Interestingly, colivelin altered this phenomenon. Moreover, following RSL3 intervention, GPX4 and SLC7A11 levels were significantly reduced, but they were elevated after hirudin treatment. Colivelin decreased their levels in the RSL3 + hirudin group (Fig. 3e). The results indicated that ferroptosis can be regulated by the STAT3/NLRP3 signaling pathway.

### Hirudin inhibited ferroptosis by downregulating the STAT3/NLRP3 signaling pathway to improve renal fibrosis in unilateral ureteral obstruction rats

**Figure 4:**
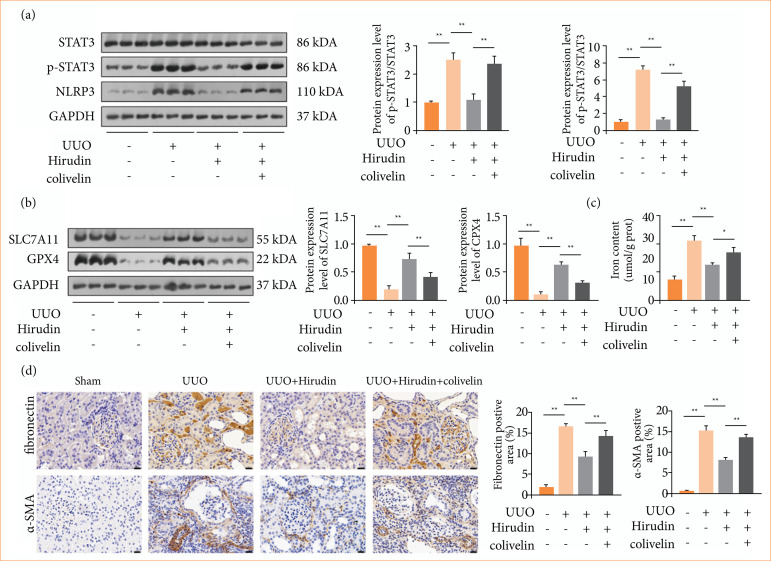
Hirudin inhibited ferroptosis by regulating the STAT3/NLRP3 signaling pathway to improve renal fibrosis in UUO rats. **(a)** Protein levels of p-STAT3 and NLRP3 were detected by Western Blot (n = 3). **(b)** Protein levels of SLC7A11 and GPX4 were detected by Western blot (n = 3). **(c)** A tissue iron assay kit was used to determine the iron content (n = 3). **(d)** α-SMA and fibronectin levels were detected by immunohistochemistry (n = 3).

To further validate the above results in vivo, we conducted experiments on UUO rats. Western blot results revealed that, after UUO induction, there was a significant increase in p-STAT3 and NLRP3 levels, accompanied by a decrease in GPX4 and SLC7A11 levels (Figs. 4a and 4b). Hirudin treatment made a significant reduction in p-STAT3 and NLRP3 expression, but an increase in GPX4 and SLC7A11 expression in UUO rats, which were reversed upon the addition of the STAT3 activator colivelin (Figs. 4a and 4b). In UUO rats, the iron content was significantly increased compared to controls (Fig. 4c). Hirudin treatment lowered the iron content in UUO rats, but colivelin restored its level (Fig. 4c). Immunohistochemistry experiments exhibited that the fibronectin and α-SMA levels were significantly elevated in UUO rats compared to controls but significantly decreased after hirudin treatment, with colivelin reversing this phenomenon (Fig. 4d). The *in-vivo* results verified that the therapeutic effect of hirudin on renal fibrosis was probably linked to the inhibition of ferroptosis via the STAT3/NLRP3 signaling pathway.

UUO: unilateral ureteral obstruction; *p < 0.05; **p < 0.01. Source: Elaborated by the authors.

## Discussion

Renal fibrosis is involved in the development of various kidney diseases, leading to detrimental changes in renal structure and function^34^. During kidney injury, invading immune cells and intrinsic kidney cells initiate an inflammatory response, releasing pro-fibrotic cytokines that promote fibrosis and ultimately lead to end-stage disease. Thus, delaying or reversing renal fibrosis and rebuilding kidney structure is essential to protect renal function. However, there are limited studies targeting renal fibrosis for treatment. In this study, the UUO rat model and RSL3-induced HK-2 cell model were established to explore potential therapies for renal fibrosis.

Hirudin is a biologically active natural compound extracted from blood-sucking leeches^16^. Hirudin was first found to have antithrombotic properties *in vitro*, and this effect may be related to the reduction of fibrin formation^35^. In recent years, the anti-inflammatory effects of hirudin have gradually emerged. Hirudin inhibits inflammation in diabetic nephropathy via the P38 MAPK/NF-κB pathway^15^. Moreover, hirudin can regulate M1 macrophage polarization and inflammation to reduce vascular damage in chronic renal failure^36^. In addition, hirudin plays a therapeutic role in CKD by reducing inflammation and preventing apoptosis of renal cells^37^.

Our results demonstrated that hirudin treatment alleviated renal injury in UUO rats and improved renal tubule dilation, lumen collapse, interstitial widening, and inflammatory cell infiltration. Additionally, renal function and renal fibrosis indicator (BUN, Crea, α-SMA, and fibronectin) levels were also decreased. These results suggested that hirudin can alleviate renal injury and inhibit epithelial-mesenchymal transition (EMT), thereby improving renal fibrosis. Similar to our results, Yu et al. found that hirudin plays an anti-fibrotic role in UUO rats^38^.

The process of ferroptosis involves ferrous ions producing reactive oxygen species (ROS) through the Fenton reaction, which causes lipid peroxidation and ultimately leads to cell membrane rupture and cell death^39^. Ferroptosis displays a crucial role in renal fibrosis, and its inhibition reduces oxidative stress, inflammation, and EMT, thereby alleviating renal fibrosis^40^^,^ 41. Tormentic acid has been shown to exert renal protective effects in UUO models by targeting inflammation, oxidative stress, and ferroptosis^42^. Consistent with this evidence, our results indicated that oxidative stress and ferroptosis levels were increased in UUO rats and RSL3-induced HK-2 cells, but they decreased after hirudin treatment. NLRP3 is a key mediator in inflammation, and pathogen- and danger-associated molecular patterns can activate NLRP3, prompting the secretion of pro-inflammatory cytokines^43^. NLRP3 has been also shown to influence ferroptosis through the Keap1-Nrf2 pathway^44^. Moreover, NLRP3 deletion ameliorates renal fibrosis by inhibiting mitochondrial dysfunction^45^. Zhang et al. found that thonningianin A improves renal interstitial fibrosis in diabetic nephropathy mice by inhibiting the NLRP3/ASC/Caspase-1 pathway^46^. Similarly, we revealed that the expression of NLRP3 was increased during the onset of renal fibrosis but decreased after hirudin treatment. Hirudin reduced inflammation and renal fibrosis, and this effect was associated with ferroptosis regulated by NLRP3.

STAT3, a member of the STAT family, mediates NLRP3 upregulation through histone hyperacetylation^47^. Additionally, STAT3 can regulate ferroptosis by decreasing GPX4 expression^48^. STAT3 is also associated with the progression of renal fibrosis, and the induction of STAT3 acetylation can promote renal fibrosis^49^^,^ 50. Research has shown that dulaglutide plays a reno-protective role in diabetic nephropathy by regulating p-STAT3 signaling, inhibiting inflammation and ferroptosis, and improving renal fibrosis^51^. Consistent with this, we found that, similar to the trend of NLRP3, p-STAT3 expression increased during ferroptosis and decreased when ferroptosis was inhibited. To further verify our hypothesis, we treated HK-2 cells and rats with the STAT3 activator colivelin and found that STAT3/NLRP3 signaling pathway and ferroptosis were activated following colivelin treatment. These findings further confirmed that the STAT3/NLRP3 signaling pathway regulates ferroptosis. Consistent with our results, studies have shown that targeting STAT3 expression can modulate the NLRP3 inflammatory pathway and ferroptosis^52^^,^ 53.

This study demonstrated that hirudin effectively treats renal fibrosis by regulating ferroptosis through the STAT3/NLRP3 signaling pathway. Nevertheless, there are some limitations. Further validation through direct inhibition of STAT3/NLRP3 pathway by genetic experiments using shRNA is necessary. Moreover, the current study only conducted experiments on cells and rats, and more research is needed to translate it into clinical application.

## Conclusion

The study demonstrated that hirudin effectively reduces ferroptosis and renal fibrosis in UUO rats and RSL3-induced HK-2 cells, while decreasing protein levels related to the STAT3/NLRP3 signaling pathway. Treatment with the STAT3 activator colivelin reversed this effect, further verifying that hirudin inhibits ferroptosis by regulating the STAT3/NLRP3 signaling pathway in renal fibrosis. Our study provided the possibility for targeted treatment of renal fibrosis.

## Data Availability

Data will be available from the corresponding author upon request.
